# The effect of cardiac resynchronization therapy on arterial stiffness and central hemodynamic parameters

**DOI:** 10.1002/joa3.12331

**Published:** 2020-03-15

**Authors:** Metin Coksevim, Murat Akcay, Serkan Yuksel, Mustafa Yenercag, Bugra Cerik, Omer Gedikli, Okan Gulel, Mahmut Sahin

**Affiliations:** ^1^ School of Medicine Department of Cardiology Ondokuz Mayis University Samsun Turkey; ^2^ Department of Cardiology Samsun Training and Research Hospital Samsun Turkey; ^3^ School of Medicine Department of Cardiology Sivas Cumhuriyet University Sivas Turkey

**Keywords:** cardiac response, cardiac resynchronization therapy, congestive heart failure, hemodynamics parameters, pulse wave velocity

## Abstract

**Introduction:**

Cardiac resynchronization therapy (CRT) is a device‐based method of treatment which decreases morbidity and mortality in heart failure with reduced ejection fraction (HFrEF). This study was aimed to investigate the effects of CRT on hemodynamic and arterial stiffness parameters evaluated by noninvasive method, and determine whether there is a correlation between the changes after CRT in these parameters and the clinical response to CRT or not.

**Methods:**

The study included 46 patients with HFrEF who were planned to undergo CRT implantation. Before the CRT implantation, clinical and demographic data were recorded from all patients. Hemodynamic and arterial stiffness parameters were measured oscillometrically by an arteriograph before CRT implantation. The patients were re‐evaluated minimum three months after CRT; the above‐mentioned parameters were measured again and compared to the pre‐CRT period.

**Results:**

Compared to the period before CRT, mean systolic blood pressure (SBP) (116.8 ± 19.1 mm Hg vs 127.7 ± 20.9 mm Hg, *P* = .005), central SBP (cSBP) (106.2 ± 17.3 mm Hg vs 116.8 ± 18.7 mm Hg, *P* = .015), cardiac output (CO) (4.6 ± 0.8 lt/min vs 5.1 ± 0.8 lt/min, *P* = .002), stroke volume (65.6 ± 16.3 mL vs 72.0 ± 14.9 mL), and pulse wave velocity (PWV) (10 ± 1.6 m/sec vs 10.4 ± 1.8 m/sec, *P* = .004) increased significantly in post‐CRT period. In addition, the same parameters were significantly increased post‐CRT period in patients with clinical response. However, there was not any similar increase in nonresponder patients.

**Conclusion:**

This study demonstrated that SBP, CO, and PWV increased significantly after CRT. The modest increases in these parameters were observed to be associated with positive clinical outcomes.

## Graphical abstract

Hemodynamic effects of CRT evaluated by non‐invasive oscillometric method and association between the clinical response.This study demonstrated that SBP, CO and PWV increased significantly after CRT. The modest increases in these parameters were observed to be associated with positive clinical outcomes.

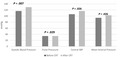

## 1 INTRODUCTION

Heart failure (HF) is a frequently seen clinical syndrome. Approximately 1%‐2% of the adult population in developed countries have HF, with the prevalence rising to ≥10% among persons 70 years of age or older.^1^ Heart failure can be classified into three types based on the condition of left ventricular (LV) systolic function: HF with preserved ejection fraction (EF) (EF ≥50%), mid‐range HF (EF: 40%‐49%) and HF with reduced ejection fraction (HFrEF) (EF <40%).^2^ In HFrEF, CRT which can be applied in selected patients in addition to medical therapy has evidence‐based positive effects both on symptoms and prognosis.^3^

Arterial stiffness or reduced aortic distensibility has an important role in patients with HF, and is valuable in prognosis.^4^, ^5^, ^6^, ^7^, ^8^ Pulse wave velocity (PWV) is the gold standard method of arterial stiffness measurement.^9^ PWV is usually measured using carotid‐femoral and brachial‐ankle methods.^9^ Many clinical studies have shown that PWV measurement can predict the clinical prognosis in cardiovascular diseases.^10^, ^11^, ^12^, ^13^ However, recently, more feasible PWV measurement became possible with the use of ankle blood pressure monitorization devices.^14^, ^15^

When contraction synchronization was provided by CRT, improvement in hemodynamic parameters such as cardiac output and a modest increase in systolic blood pressure (SBP) and pulse pressure (PP) is expected.^16^, ^17^, ^18^ However, the impact of CRT on PWV has yet to be investigated. Our study aimed to evaluate the effect of CRT on hemodynamic parameters and PWV by measuring these parameters using a noninvasive, oscillometric method. Additionally, we aimed to detect whether there was an association between these parameters and clinical response to CRT.

## 2 METHODS

### 2.1 Study population

This prospective study included 46 patients (76.1% males (n = 35), 23.9% females (n = 11) who admitted to Ondokuz Mayis University, Faculty of Medicine, Cardiology Department between November 2015 and September 2017, have indication for CRT implantation according to the 2016 European Society of Cardiology guideline for the diagnosis and treatment of acute and chronic HF. CRT implantation was performed to all participants. The study excluded patients with acute heart failure, life expectancy less than one year due to a systemic disease, valvular heart disease requiring intervention, atrial fibrillation, change in medical therapy of heart failure during follow‐up, severe pulmonary hypertension (>60 mm Hg), acute renal and/or hepatic failure, hypo‐/hyperthyroidism, severe pulmonary diseases, and fistula, aneurysm or stenosis in brachial artery region.

Age, sex, body weight, height, body mass index (BMI), cardiovascular risk factors, medications and treatment compliance, and other comorbidities were recorded for all patients. New York Heart Association (NYHA) functional class, 6‐minute walk test (6MWT), electrocardiographic findings, laboratory and echocardiographic data, arterial stiffness measurements and hemodynamic parameters were recorded. Study patients were evaluated again minimum 3 months after the CRT implantation. All included patients gave written informed consents. In addition, the ethics committee approval for the study was granted by Ondokuz Mayis University, Faculty of Medicine and Ethics Committee with the decision numbered OMU KAEK 2016/90.

### 2.2 The measurement of arterial stiffness and hemodynamic parameters

Mobil‐O‐Graph 24 hours ABPM NG® arteriograph was used to measure arterial stiffness and cardiovascular hemodynamic parameters. All measurements were performed by a single experienced operator (medical doctor) who was blinded to other data. Each subject rested in a seated position for 10 minutes in a quiet room at 20°C‐23°C before the baseline hemodynamic measurements were recorded. Brachial blood pressure (BP) and heart rate (HR) were measured in the right arm before using arteriograph. Three sequential measurement sessions, separated by a 5‐min interval were obtained. A total of six measurements, one of which was a control, were taken automatically by the device for a session. The mean values of each session were recorded. If the mean PWV difference between sessions was less than 0.5 m/sec, the mean was used for the analysis. Otherwise, a third measurement session was to be conducted and the median of the three measurements was calculated. Thus, biases caused by single investigator measurements were eliminated. Also the measurements at follow‐up were performed at the same time of the day to minimize the variation as proposed by consensus.^19^

**Figure 1:**
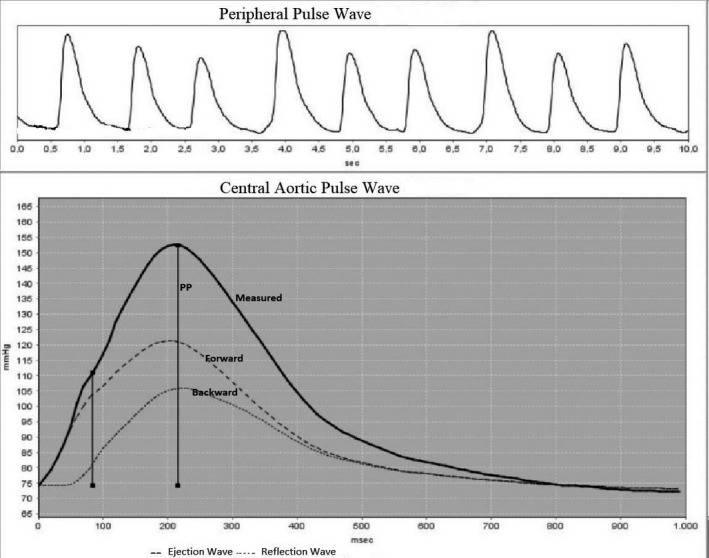
Evaluation of aortic pulse wave velocity using a transfer function from brachial pressure wave analysis

The measurement was performed after proper cuff size was selected (two sizes available: 24‐34 and 32‐42 cm). Briefly, underlying working principle of the oscillometric method is; to record oscillometric pressure curves based on plethysmography and register pulsatile pressure changes in an artery on the upper arm. After the conventional oscillometric BP measurement, the cuff instantly re‐inflates and the pulse waves are recorded at diastolic blood pressure (DBP) level for 10 seconds. By this method, the central aortic pressure is calculated from the brachial BP using a transfer function (TF) and the central aortic waveform is decomposed into forward and reflected waves using an uncalibrated triangular aortic flow waveform. The idea behind a TF is the modification of a certain frequency range within the acquired pulse signal to derive the aortic pressure wave (ARCSolver algorithm). The waves formed by central pressure changes amplified and transferred to device‐specific tonometry and uploaded to HMS Client Server 5.1® software which was developed specifically for the device. Mobil‐O‐Graph 24 hours PWA Monitor uses these mechanisms for obtaining measurements which are validated.^14^, ^15^, ^20^, ^21^ (Figure 1).

### 2.3 Echocardiography

Transthoracic echocardiography (TTE) was performed by an experienced echocardiography specialist who was blinded to other data. Vivid E9 (GE Vingmed Ultrasound, Horten, Norway) TTE device and M5S (1.5‐4.5 MHz) ultrasound probe were used for the echocardiographic measurements. Left ventricle end‐diastolic (LVEDD) and end‐systolic diameters (LVESD), and left atrium (LA) anteroposterior diameter were measured from the long‐axis view of the heart using TTE. Ejection fraction (EF) was calculated by Modified Simpson method using apical 4‐chamber and 2‐chamber images. Valvular heart pathologies were detected and graded. Pulmonary arterial pressure was measured.

### 2.4 CRT implantation

Following left pectoral region incision, subclavian venous puncture was performed, and right ventricle and right atrium leads were placed. After this, coronary sinus was found using a left amplatz catheter, and images were recorded using a contrast‐enhancing agent for the selection of the suitable branch. Left ventricular (LV) lead was placed on the lateral or posterolateral branch of coronary sinus if possible. All electrodes were connected to the generator, and the pouch was closed following stimulus and threshold values were controlled***.*** After implantation AV delay of the patients was set to be 120 ms, and VV delay was 0 ms for optimal resynchronization.

### 2.5 Follow‐up the patients

The patients were invited for follow‐up minimum three months after CRT implantation. The mean follow‐up period of the patients was 20 weeks. They were questioned for their compliance to medical therapy and whether they were hospitalized for HF. The information whether they had a dose change in their medications during follow‐up period was noted. Their body weights and heights were measured again, and the BMI was re‐calculated. Their functional capacities were evaluated according to the NYHA classification, 6MWT was performed. Electrocardiography (ECG) was taken. Arterial stiffness and cardiovascular hemodynamic parameters were measured again. Pacemaker was controlled, and the ventricular pace rate being 95% and/or above was recorded.

### 2.6 Definitions

Experts consider aortic pulse wave velocity as the gold standard in the assessment of arterial stiffness.^9^ Therefore, we considered PWV as a primary arterial stiffness marker in our study.

Ischemic cardiomyopathy and nonischemic cardiomyopathy definitions were made based on the presence or absence of myocardial infarction events or 75% or more stenosis in the left coronary artery.

Patients with 50‐meter and/or 20% increase in 6MWT after CRT, and who were not hospitalized due to decompensation of heart failure during the follow‐up were considered as “clinical responders.” Those who do not meet these criteria considered as "nonresponders."

### 2.7 Statistical analysis

Study data were computerized using “SPSS (Statistical Package for Social Sciences) for Windows 21.0 (SPSS Inc)” and assessed. Descriptive statistics were expressed as mean ± standard deviation, frequency distribution, and percentage. The distribution normality of the variables was examined using visual (histogram and probability graphs) and analytic methods (Shapiro‐Wilk Test***).*** For the normally distributed variables Student's T Test was used for the statistical significances between two independent groups, and Paired Sample T Test for the statistical significances between two dependent groups. For not normally distributed data, as statistical methods Mann‐Whitney U Test were used between two independent groups and Wilcoxon signed‐rank test were used between two dependent groups. Statistical significance level was considered as *P* value < .05.

## 3 RESULTS

The mean age of 46 patients investigated in the study was 70.6 ± 7.9 (56‐85 year) years with 76.1% of them being males. While 45.7% of the patients had ischemic HF and 54.3% had nonischemic HF. The mean HF duration of the patients was 8.0 ± 4.00 (3‐20 year) years. As for the comorbidities, 56.5% of 46 patients had hypertension (HT), 26.1% had diabetes mellitus (DM), and 19.6% had hyperlipidaemia. 32.6% of the patients were smokers, and all patients were receiving optimal medical therapy (including beta‐blocker, ACEI/ARB, spironolactone). 54.3% of the patients under assessment were in NYHA class II, 32.6% were in NYHA class III, and 13.0% were in NYHA class III/IV. The mean LVEF of the patients was 28.1% (Table 1). Table 1 showed other demographic and baseline clinical characteristics of patients.

**Table 1:** Basic demographic and clinical characteristics of the patients included in the study

Heart failure patients (n = 46)	Value
Age (year), mean ± SD (min‐max)	70.6 ± 7.9 (56‐85)
Gender, n (%)	
Male	35 (76,1)
Female	11 (23,9)
Height (m), mean ± SD (min‐max)	1.7 ± 0.1 (1.5‐1.8)
Weight (kg), mean ± SD (min‐max)	73.4 ± 13.4 (51‐105)
BMI (kg/m^2^), mean ± SD (min‐max)	26.6 ± 4.4 (19.6‐37.1)
Etiology of heart failure, n (%)	
Non‐ischemic	25 (54,3)
Ischemic	21 (45,7)
Duration of heart failure (year), mean ± SD (min‐max)	8.0 ± 4.0 (3‐20)
Diabetes mellitus, n (%)	12 (26,1)
Hypertension, n (%)	26 (56,5)
Hyperlipidemia, n (%)	9 (19,6)
Smoking, n (%)	15 (32,6)
Optimal medical therapy, n (%)	46 (100)
NYHA Class, n (%)	
II	25 (54,3)
III	15 (32,6)
III/IV	6 (13,0)
ECG (branch block), n (%)	
Left bundle branch block (LBBB)	38 (82,6)
Other branch blocks	8 (17,4)
GFR (mL /min/1,73 m^2^), mean ± SD (min‐max)	62.5 ± 20.6 (21.8‐98.1)
Basal echocardiography parameters, mean ± SD (min‐max)	
LVEDD (mm)	61.8 ± 9.8 (42‐91)
LVESD (mm)	52.7 ± 10.3 (32‐81)
LA (anterior‐posterior diameter) (mm)	45.0 ± 5.0 (30‐54)
Ejection fraction (%)	28.1 ± 5.0 (16‐36)
LVOT VTI	13.3 ± 3.7 (6.7‐21.8)

Abbreviations: BMI, Body Mass Index; GFR, Glomerular filtration index; LA, Left atrium; LVEDD, Left ventricular end diastolic diameter; LVESD, Left ventricular end systolic diameter; LVOT VTI, Left ventricular outflow tract velocity time index; n, Number of patients; NYHA, New York Heart Association; SD, Standard deviation; %, Percent.

### 3.1 Results during the follow‐up

32.6% of the patients were hospitalized due to HF decompensation and two patients (4.3%) died during the follow‐up period. One patient died due to septic shock after pneumonia and the other patient died due to retroperitoneal bleeding caused by warfarin overdose.

**Figure 2:**
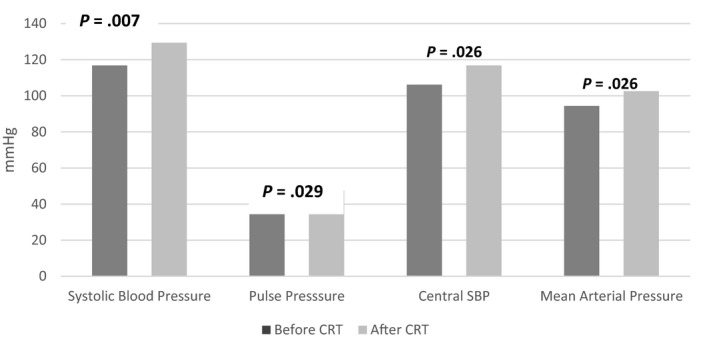
Change in arterial pressure before and after CRT

**Figure 3:**
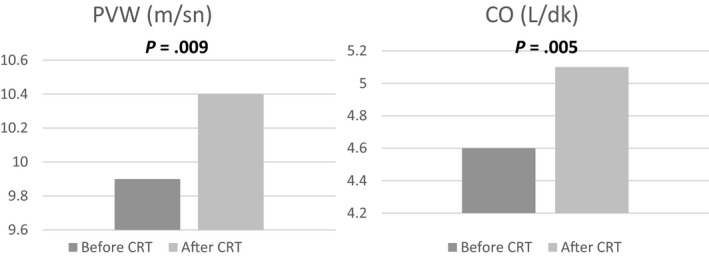
Changes in PWV and CO before and after CRT

Table 2 shows the clinical findings and arterial stiffness parameters (PWV and augmentation index [AIx]) before and minimum 3 months after the CRT implantation, and the changes in hemodynamic parameters. Heart rate was similar between two groups. Post‐CRT 6MWT was significantly increased compared to pre‐CRT period, and QRS duration was significantly decreased (*P* < .05). When cardiovascular hemodynamic parameters were assessed, post‐CRT SBP, mean arterial pressure (MAP), PP, cSBP, stroke volume, and CO were significantly increased compared to pre‐CRT values (*P* < .05). Also, PWV was significantly increased compared to pre‐CRT values (*P* < .05), no statistically significant change was observed in AIx (*P* > .05) (Table 2; Figures 2 and 3).

**Table 2:** Evaluation of arterial stiffness parameters before and after cardiac resencronization therapy

Heart failure patients (n = 44)	Pre CRT	Post CRT	*P* value*
	mean ± SD	mean ± SD	
Six minute walking test (m)	217.1 ± 118.6	323.8 ± 152.5	<.001
Heart rate (bpm/min)	71.1 ± 17.4	75.5 ± 11.8	.099
QRS duration (msn)	150.0 ± 18.3	115.7 ± 25.8	<.001
Augmentation index (Alx) (%)	22.2 ± 13.5	18.0 ± 10.2	.184
Pulse wave velocity (PWV) (m/sn)	9.9 ± 1.5	10.4 ± 1.8	.009
Systolic blood pressure (SBP) (mm Hg)	116.8 ± 19.1	129.4 ± 20.6	.007
Diastolic blood pressure (DBP) (mm Hg)	75.4 ± 13.3	80.1 ± 10.1	.145
Mean arterial pressure (MAP) (mm Hg)	94.4 ± 14.9	102.6 ± 13.6	.026
Pulse pressure (PP) (mm Hg)	41.2 ± 13.3	47.7 ± 14.8	.029
central SBP (mm Hg)	106.2 ± 17.3	116.8 ± 18.7	.026
central DBP (mm Hg)	77.2 ± 12.6	81.2 ± 10.8	.232
Cardiac output (CO) (lt/min)	4.6 ± 0.8	5.1 ± 0.9	.005
Stroke volume (mL)	65.6 ± 16.3	72.0 ± 14.9	.027

Abbreviations: N, number of patients; SD, Standard deviation.

* Paired Sample T Test.

**Figure 4:**
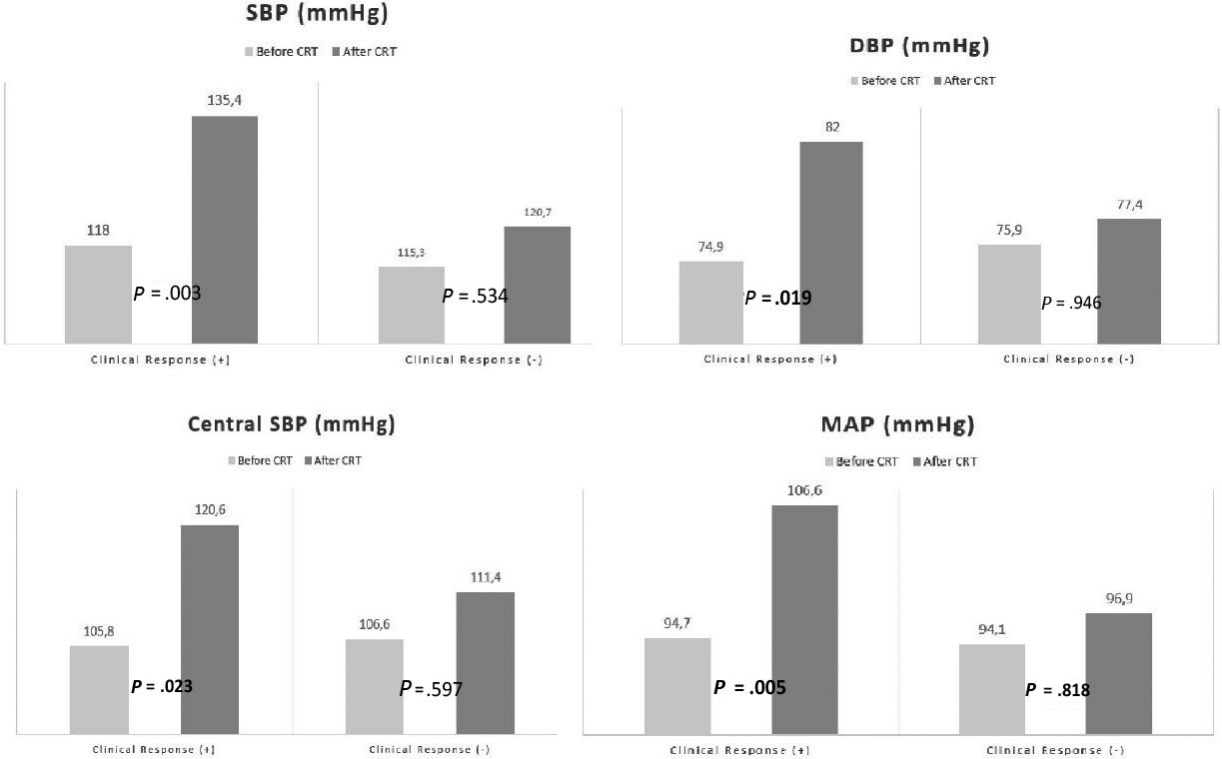
Changes in arterial pressure of patients with and without clinical response after CRT

**Figure 5:**
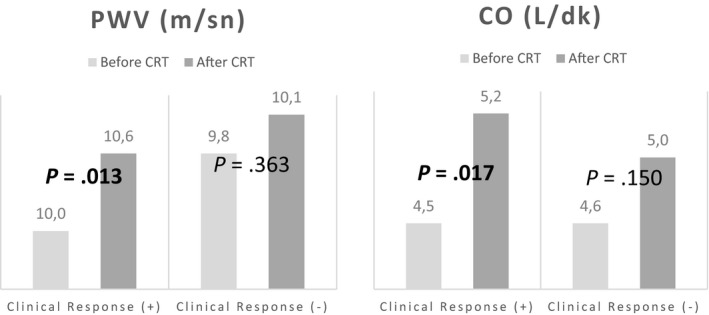
Changes in PWV and CO of patients with and without clinical response after CRT

Of 44 participants who survived within the first 3 months after CRT, 56.8% (n = 25) had clinical response and 43.2% (n = 19) did not. Table 3 showed demographic and baseline clinical characteristics of responder and nonresponder patients. Table 4 shows the distribution of 6MWT, QRS duration, cardiovascular hemodynamic and arterial stiffness parameters of the responders and nonresponders. When intra‐group assessment was performed for the clinical responders; post‐CRT SBP, DBP, MAP, cSBP, cDBP CO, and PWV were significantly increased compared to pre‐CRT values (*P* < .05). No statistically significant difference was detected in the same parameters for nonresponders (*P* > .05) (Table 4; Figures 4 and 5).

**Table 3:** Distribution of basic demographic and clinical characteristics of the patients with and without clinical response

	Clinical Response (+) (n = 25)	Clinical Response (‐) (n = 19)	*P* value
Age (year), mean ± SD (min‐max)	70.6 ± 8.1 (59‐85)	71.3 ± 8.7 (56‐88)	.797*
Gender, n (%)			
Male	19 (76.0)	14 (73.7)	1.000***
Female	6 (24.0)	5 (26.3)	
BMI (kg/m^2^), mean ± SD (min‐max)	26.1 ± 3.8 (19.6‐33.3)	27.6 ± 5.4 (19.6‐37.5)	.309*
Etiology of heart failure, n (%)			
Non‐ischemic	15 (60.0)	8 (42.1)	.239**
Ischemic	10 (40.0)	11 (57.9)	
Duration of heart failure (year), mean ± SD (min‐max)	8.2 ± 4.1 (3‐15)	7.5 ± 4.1 (2‐20)	.594*
Diabetes mellitus, n (%)	6 (24.0)	6 (31.6)	.576**
Hypertension, n (%)	12 (48.0)	14 (73.7)	.086**
Hyperlipidemia, n (%)	4 (16.0)	5 (26.3)	.467***
Smoking, n (%)	9 (36.0)	5 (26.3)	.495**
NYHA class, n (%)			
II	20 (80.0)	5 (26.3)	
III	4 (16.0)	9 (47.4)	.001**
III/IV	1 (4.0)	5 (26.3)	
ECG (branch block), n (%)			
LBBB	20 (80.0)	16 (84.2)	1.000**
Other branch blocks	5 (20.0)	3 (15.8)	
GFR (mL/min/1,73 m^2^), mean ± SD (min‐max)	69.8 ± 18.5 (32.6‐98.1)	53.7 ± 20.7 (21.8‐97.0)	.010*

Abbreviations: BMI, Body Mass Index; GFR, Glomerular filtration index; n, Number of patients; NYHA, New York Heart Association; SD, Standard deviation; %, Percent.

* Student's T Test.

** Chi‐Square Test.

*** Fisher's Exact Test.

**Table 4:** Distribution of arterial stiffness parameters of patients with and without clinical response

	Clinical Response (+) (n = 25)	Clinical Response (‐) (n = 19)	*P* value*
	mean ± SD	mean ± SD	
QRS duration (msn)			
Pre CRT	145.5 ± 11.6	155.6 ± 23.4	.099
Post CRT	108.8 ± 24.2	125.7 ± 25.5	.058
*p* **	<0.001	0.002	
Augmentation index (%)			
Pre CRT	23.8 ± 13.2	20.2 ± 14.1	.442
Post CRT	18.8 ± 9.4	16.9 ± 11.6	.602
*p* **	0.127	0.962	
Pulse wave velocity (m/sn)			
Pre CRT	10.0 ± 1.5	9.8 ± 1.6	.685
Post CRT	10.6 ± 1.9	10.1 ± 1.6	.466
*p* **	0.013	0.363	
SBP (mm Hg)			
Pre CRT	118.0 ± 17.4	115.3 ± 21.6	.687
Post CRT	135.4 ± 21.4	120.7 ± 16.5	.038
*p* **	0.003	0.534	
DBP (mm Hg)			
Pre CRT	74.9 ± 12.0	75.9 ± 15.2	.822
Post CRT	82.0 ± 9.4	77.4 ± 10.8	.188
*p* **	0.019	0.946	
MAP (mm Hg)			
Pre CRT	94.7 ± 13.3	94.1 ± 17.2	.905
Post CRT	106.6 ± 13.4	96.9 ± 12.3	.041
*p* **	0.005	0.818	
Pulse pressure (mm Hg)			
Pre CRT	43.2 ± 13.4	38.8 ± 13.3	.327
Post CRT	51.4 ± 16.0	42.5 ± 11.5	.070
*p* **	0.054	0.321	
Central SBP (mm Hg)			
Pre CRT	105.8 ± 16.0	106.6 ± 19.4	.905
Post CRT	120.6 ± 20.3	111.4 ± 15.3	.162
*p* **	0.023	0.597	
Central DBP (mm Hg)			
Pre CRT	76.0 ± 12.6	78.8 ± 12.7	.515
Post CRT	83.0 ± 10.5	78.8 ± 11.2	.278
*p* **	0.037	0.704	
Cardiac output (lt/min)			
Pre CRT	4.5 ± 0.8	4.6 ± 0.7	.695
Post CRT	5.2 ± 1.0	5.0 ± 0.7	.669
*p* **	0.017	0.150	
Stroke volume (mL)			
Pre CRT	65.0 ± 16.2	66.3 ± 16.9	.821
Post CRT	71.1 ± 12.9	73.4 ± 17.8	.661
*p* **	0.058	0.240	
Heart rate (bpm/min)			
Pre CRT	70.0 ± 18.5	72.4 ± 16.4	.690
Post CRT	76.2 ± 10.6	74.7 ± 13.4	.717
*p* **	0.145	0.470	

Abbreviations: CRT, cardiac resynchronization therapy; n, number of patients; SD, Standard deviation.

* Student's T Test.

** Paired Sample T Test.

## 4 DISCUSSION

This study showed that CRT increased SBP, PP, and CO using a noninvasive oscillometric method. In addition, while we observed a significant increase in these parameters in patients that clinically responded to CRT, we did not observe a comparable increase in nonresponding patients.

There is a close association between HF and arterial blood pressure. The lower BP in patients with reduced EF leads to worse outcomes, and higher BP leads to more positive outcomes.^22^, ^23^, ^24^, ^25^, ^26^ By establishing contraction resynchronization, CRT aims to increase the LV pump activity, ventricular filling and EF, and decrease the LVESD and LVEDD, and if present, the severity of mitral insufficiency. Thereby, ventricular remodelling can be halted and, in fact, reversed.^27^

Although there is strong evidence that CRT increases EF and CO, the number of studies showing its effect on BP is limited. While a modest increase was detected in SBP after CRT in previous studies,^16^, ^17^, ^18^ a few studies have associated the baseline BP values and clinical response.^18^ In their study, Biton et al shown that the patients with increased SBP after CRT tend to have lower mortality compared to the patients with unchanged or decreased BP.^28^

As a result of these data, it is expected that CRT will increase the CO and stroke volume in patients with HFrEF, thereby increasing BP. Concordantly we detected a significant increase in SBP due to increase in CO and stroke volume after CRT in the whole study population. We detected the same increase in patients with positive clinical response to CRT, but not in those without clinical response. And also in post‐CRT period, there was a statistically significant difference in SBP and MAP between two groups. So we considered that a moderate increase in BP might affect the positive response to CRT. Furthermore, this increase in SBP after CRT would provide an opportunity to continue or increase the dosage of the standard, life‐extending HF medications, thereby, to contribute the better outcomes. However, it is obvious that larger studies are needed to prove whether arterial pressure increase improves clinical outcomes after CRT implantation.

The prognostic importance of arterial stiffness was investigated in many cardiovascular diseases including HF.^29^ Arterial stiffness is affected by many factors, namely HT, DM, coronary artery disease, peripheral vascular disease, chronic renal failure, and medications; and the controversy around how it would affect the reduced heart pumping functions still remains.^30^ Arterial stiffness can be assessed using noninvasive methods such as Alx and PWV in addition to many other methods.^9^ PWV is one of the most widely used, and is considered to be more reliable especially in HF patients compared to wave analysis measurements such as AIx. As individuals with impaired LV function, PP and AIx are not reliable measures of wave reflection. In contrast, PWV, as a measure of central aortic stiffness, is relatively less confounded by LV dysfunction.^31^

Arnold et al have found a decrease in arterial compliance in their study with. HFrEF patients regardless of the HF etiology. They thought that decreased arterial compliance would contribute to the pathophysiology of HF by increasing the LV wall stress.^32^ Patrianakos et al evaluated arterial stiffness in patients with HFrEF. This study compared 60 patients (mean LVEF of ~40%) with healthy controls, and found that the proximal aortic stiffness calculated by echocardiographic method was higher in the HF group.^33^ Demir et al evaluated 98 patients with ischemic cardiomyopathy and found that a PWV cut‐off value of 11.06 m/sec was a predictor for mortality in these patients.^6^ Regnault et al investigated 306 patients in EPHESUS study, and showed that increased PWV contributed to major adverse cardiovascular outcomes. In their study, mean PWV was above 11 m/sec.^34^ In our study, the mean PWV of the patients was 9.9 ± 1.5 m/sec. After CRT, it increased to 10.4 ± 1.8 m/sec (*P* = .009). Even though PWV increased following CRT, it is important to note that it was not above the values that were shown to be associated with poor prognosis in the literature.^35^

Up to 30%‐45% of patients treated with CRT do not clinically respond.^36^ Despite many potential predictors were investigated for evaluating the response to CRT, none were found to be reliable except QRS duration and morphology (Left bundle brunch block [LBBB]), HF etiology (nonischemic), gender (female), and rhythm (sinus rhythm).^37^ When defining response to CRT, clinical measures such as NYHA class and quality of life measurements, 6MWT, exercise duration and metabolic exercise tests can be used.^38^ In our study, we obtained clinical response in more than 50% of patients in accordance with the literature. Although QRS durations were wider, patients with Non‐LBBB QRS morphology were less likely to respond to CRT. PWV was not significantly different in patients with and without clinical response. Therefore, our study could not suggest that PWV could be used as a predictor for response to CRT.

There is no study in the literature evaluating the post‐CRT PWV change and its association with clinical response. We detected a statistically significant increase in PWV 3 months after CRT. Also we observed an increase in PWV in patients with clinical response, however, did not observe the same increase in patients without clinical response. Although increase in PWV is known to contribute to adverse clinical outcomes for cardiovascular diseases, it is not likely that CRT which was proved to increase CO, stroke volume, SBP and PP would cause a decrease in PWV. It is hard to say that the improvement of PWV independently predicts favourable CV outcome. This increase in PWV is thought to be a reflection of increased systolic blood pressure rather than an independent outcome. Further studies needed to confirm our findings. According to the data obtained in our study, modest increases in CO, SBP and PWV seen after CRT appear to be associated with positive clinical outcomes.

## 5 STUDY LIMITATIONS

Several limitations have to be acknowledged. Firstly, short follow‐up duration, lower statistical power and results were major limitations. Also the patients included were not grouped by the etiology of heart failure. Even though patients were receiving optimal medical therapy and did not have dose modification during the follow‐up, not all patients were being treated with the same dose and medications. Lastly, this is a single center study and the measurements were made by a single researcher.

## 6 CONCLUSION

The systolic blood pressure, cardiac output, stroke volume, and PWV were increased after cardiac resynchronization therapy. The modest increases in these parameters appeared to be associated with positive clinical outcomes. The efficacy of CRT can be assessed easily by noninvasive measurement of these parameters.

## CONFLICT OF INTEREST

The authors declare no conflict of interest.
